# Implementation of eHealth to Support Assessment and Decision-making for Residents With Dementia in Long-term Care: Systematic Review

**DOI:** 10.2196/29837

**Published:** 2022-02-03

**Authors:** Juliet Gillam, Nathan Davies, Jesutofunmi Aworinde, Emel Yorganci, Janet E Anderson, Catherine Evans

**Affiliations:** 1 Cicely Saunders Institute King's College London London United Kingdom; 2 Centre for Ageing Population Studies, Research Department of Primary Care and Population Health University College London London United Kingdom; 3 Centre for Dementia Palliative Care Research, Marie Curie Palliative Care Research Department University College London London United Kingdom; 4 School of Health Sciences City, University of London London United Kingdom; 5 Sussex Community National Health Service Foundation Trust Brighton General Hospital Brighton United Kingdom

**Keywords:** telemedicine, implementation science, dementia, long-term care, systematic review

## Abstract

**Background:**

As dementia progresses, symptoms and concerns increase, causing considerable distress for the person and their caregiver. The integration of care between care homes and health care services is vital to meet increasing care needs and maintain quality of life. However, care home access to high-quality health care is inequitable. eHealth can facilitate this by supporting remote specialist input on care processes, such as clinical assessment and decision-making, and streamlining care on site. How to best implement eHealth in the care home setting is unclear.

**Objective:**

The aim of this review was to identify the key factors that influence the implementation of eHealth for people living with dementia in long-term care.

**Methods:**

A systematic search of Embase, PsycINFO, MEDLINE, and CINAHL was conducted to identify studies published between 2000 and 2020. Studies were eligible if they focused on eHealth interventions to improve treatment and care assessment or decision-making for residents with dementia in care homes. Data were thematically analyzed and deductively mapped onto the 6 constructs of the adapted Consolidated Framework for Implementation Research (CFIR). The results are presented as a narrative synthesis.

**Results:**

A total of 29 studies were included, focusing on a variety of eHealth interventions, including remote video consultations and clinical decision support tools. Key factors that influenced eHealth implementation were identified across all 6 constructs of the CFIR. Most concerned the inner setting construct on requirements for implementation in the care home, such as providing a conducive learning climate, engaged leadership, and sufficient training and resources. A total of 4 novel subconstructs were identified to inform the implementation requirements to meet resident needs and engage end users.

**Conclusions:**

Implementing eHealth in care homes for people with dementia is multifactorial and complex, involving interaction between residents, staff, and organizations. It requires an emphasis on the needs of residents and the engagement of end users in the implementation process. A novel conceptual model of the key factors was developed and translated into 18 practical recommendations on the implementation of eHealth in long-term care to guide implementers or innovators in care homes. Successful implementation of eHealth is required to maximize uptake and drive improvements in integrated health and social care.

## Introduction

### Background

Dementia is a progressive, complex neurodegenerative condition with a multitude of types and clinical presentations. It is the leading cause of death in the United Kingdom [1] and is projected to have the highest global proportional rise (264%) in suffering associated with a need for palliative care [2]. Dementia is characterized by a complexity of care needs, which advance as the condition progresses [3]. These needs span multiple domains of health care, and multimorbidity is common [4]. Symptoms such as pain and breathlessness [5,6] cause significant distress for the person and their caregiver and increase toward the end of life [7].

Over half of the people with dementia (58%) die in care homes in England [8]. They are the main providers of end-of-life dementia care, with the average life expectancy on admission for a resident with dementia being 1 to 2 years [9]. The term *care home* in the United Kingdom refers to both residential and nursing homes. These differ with regard to the provision of input from health care professionals, with nursing homes providing additional access to 24-hour on-site nursing care. Care homes require the resources to deliver multidisciplinary care to meet these advancing and acute dementia-specific needs [10]. Providing access to good quality, continuous care throughout the dementia trajectory is essential. This can be achieved by integrating care homes with primary care, palliative care, and dementia care teams to enable multidisciplinary and specialist input on vital care processes [11].

Care needs change and develop over time and cause considerable distress if left unmet [6]. A comprehensive assessment by a multidisciplinary team to review medical, functional, mental, and social abilities is essential for this population with complex needs [12]. Interprofessional collaboration is also required to share clinical expertise and experience, best inform complex clinical decisions concerning treatment for multimorbidities, and deliver appropriate care [13]. Integrating these processes across services is widely acknowledged to improve person-centered treatment outcomes for older people with complex needs [11] and reduce detrimental transitions between settings occurring in the final years of life, such as unplanned hospitalization [14].

To integrate services and deliver continuous and coordinated care, established methods of communication are required to share information between systems about residents’ care needs and outcomes. A way to facilitate this is through the use of eHealth [15], which is defined as “health services and information delivered or enhanced through the internet and related technologies” [16], encompassing an array of interventions that enable care to be delivered remotely.

The COVID-19 pandemic has had major implications for the use of eHealth in care homes. In response, former barriers to the integration of health and social care services in England have been liberalized, with restrictions on information sharing between services relaxed [17]. The pandemic highlighted the lack of systems for efficiently sharing information between services nationally and internationally [18,19]. This compromised the delivery of care to meet the escalating health care needs and residents’ quality of life. Innovation was required to communicate between services for the comprehensive assessment of symptoms and management of care and outcomes. eHealth provides a way to do this [20].

### Objective

There is little evidence to guide the design and implementation of eHealth resources to manage symptoms and concerns for people with dementia in care homes. The key task at hand is to understand how we can scale-up eHealth interventions to embed them in routine care. Despite positive findings regarding their benefit [21], eHealth interventions are yet to attain widespread implementation in care homes, and the way of effectively achieving this remains unclear. This study aimed to explore factors that influence the implementation of eHealth interventions to support assessment and decision-making regarding care and treatment for people with dementia in care homes.

## Methods

### Design

The review is reported in accordance with the PRISMA (Preferred Reporting Items for Systematic Reviews and Meta-analyses; Multimedia Appendix 1) guidelines. A systematic review, using narrative synthesis and thematic analysis, was conducted to identify common facilitators of and barriers to eHealth implementation in care homes. The synthesis followed the guidance of Popay et al [22], which provides a specific direction for reviews concerned with the implementation of interventions. The protocol for this review was registered on PROSPERO (International Prospective Register of Systematic Reviews; registration number CRD42020184587). Before registering this review, a search of PROSPERO was conducted to ensure that no similar reviews were underway. Our final search was performed in the week the review was registered (June 1, 2020). Originally, the scope of the review also included collecting data on intervention effectiveness and key components; however, given the breadth of evidence relating to these outcomes, the findings regarding effectiveness will be reported in a second review.

### Search Strategy

A total of 4 databases (Embase, PsycINFO, MEDLINE, and CINAHL) were searched for studies published in English from January 2000, with the final search being on April 28, 2020. The year 2000 was chosen as the *cutoff* year to exclude eHealth that may be outdated in the context of today’s technological advancements. A search strategy was developed with the help of an information support specialist and informed by scoping the literature for the types of eHealth interventions currently in use (see Multimedia Appendix 2 for the full search strategy). A combination of Medical Subject Headings terms and keywords was used to develop a strategy based on the following concepts: *dementia* AND *care homes* AND *eHealth* AND *assessment* OR *decision-making*. The search strategy was complemented through reference chaining and citation tracking using Google Scholar, following the initial identification of studies from the database search. The eligibility criteria were developed using the PICO (population, intervention, control, and outcomes) acronym, following recommendations on its suitability and enhanced sensitivity when conducting qualitative systematic reviews [23]. The criteria are outlined in Textbox 1.

**Eligibility criteria.**

**Inclusion criteria**

- Population: residents with a diagnosis of dementia residing in a long-term care facility; studies that included residents with dementia in a mixed population
- Intervention: eHealth interventions that aimed to facilitate comprehensive assessment of care home residents or improve decision-making about care and treatment; eHealth interventions that enable care coordination between practitioners and the sharing of information between settings to facilitate integrated working, such as between care homes and health care services
- Outcome: data relating to factors that influence or inhibit the implementation of eHealth interventions in care homes
- Comparator: no restrictors
- Study design: all study designs that reported data relating to implementation

**Exclusion criteria**

- Population: people with a diagnosis of dementia living at home or staying in short-term care or acute care settings; studies that did not mention dementia in the population
- Intervention: nondigitalized intervention studies; eHealth interventions that did not focus on aiding comprehensive assessment or supporting clinical decision-making; interventions that monitored clinical signs only, such as a motion sensor, or recorded biodata, for example, blood pressure remote monitoring, were out of scope
- Outcome: not applicable
- Comparator: not applicable
- Study design: opinion pieces

### Study Selection Procedure

Studies identified from the search were exported to Endnote [24]. Duplicates were identified and removed. All titles and abstracts were screened by 1 researcher (JG). Approximately 20% were randomly selected for blinded double screening by 2 independent researchers (TA and EY) as a calibration process to test the application of the eligibility criteria. Once an agreement of 90% was confirmed between the reviewers, the eligibility criteria were applied to all identified studies. This was undertaken to screen for eligibility at the title and abstract screening and again at the full paper review. Discrepancies were resolved through discussion.

### Quality Appraisal

The quality of publications was appraised using the Critical Appraisal Skills Programme (CASP) tool [25], which was appropriate for the study design. It was conducted by 1 author (JG) and reviewed independently by 2 authors (CE and ND). Where the design was not amenable to the CASP, alternative tools were used, including the Mixed Methods Appraisal Tool (MMAT) [26] and the Joanna Briggs Institute (JBI) critical appraisal tools [27]. No studies were excluded only on the basis of their appraised quality; rather, it was conducted to help understand and describe the studies.

### Data Extraction

A standardized data extraction tool was developed in Microsoft Excel and informed by the review questions. Extracted data included study aim, country of origin, design, population of interest, setting, eHealth intervention (type, components, and summary), methods of data collection and analysis, outcomes regarding intervention implementation and conclusions, implications, and limitations. Implementation data were extracted from both the results and discussion sections to capture relevant findings relating to the authors’ observations of why an intervention was or was not effective.

### Data Analysis and Synthesis

A deductive thematic analytic approach was undertaken, underpinned by the adapted version of the Consolidated Framework for Implementation Research (CFIR) [28]. The original CFIR [29] comprises 5 broad theory-based constructs and 39 subconstructs within these: intervention characteristics, inner setting, outer setting, individual characteristics, and implementation process. It is a comprehensive and practical guide for assessing the potential factors that influence implementation. A recent adaptation of the framework added a sixth construct [28], *patient needs*, acknowledging the importance of person-centered care in health care interventions and the paucity of attention it frequently receives in implementation frameworks. Given the nature of this review, the adapted CFIR was used.

Preliminary synthesis involved tabulation to organize the findings and compare data across different studies. The data from each study were then mapped onto the framework. Common themes that arose across the studies in line with the subconstructs of the framework were then synthesized to form a narrative regarding facilitating elements of and inhibiting barriers to successful implementation. Where data did not align to a subconstruct, an inductive thematic analytic approach was undertaken to avoid biasing data and identify gaps in the framework when applying it to this context. The themes were then developed to identify additional constructs to adapt the framework to this specific context.

## Results

### Summary

**Figure 1:**
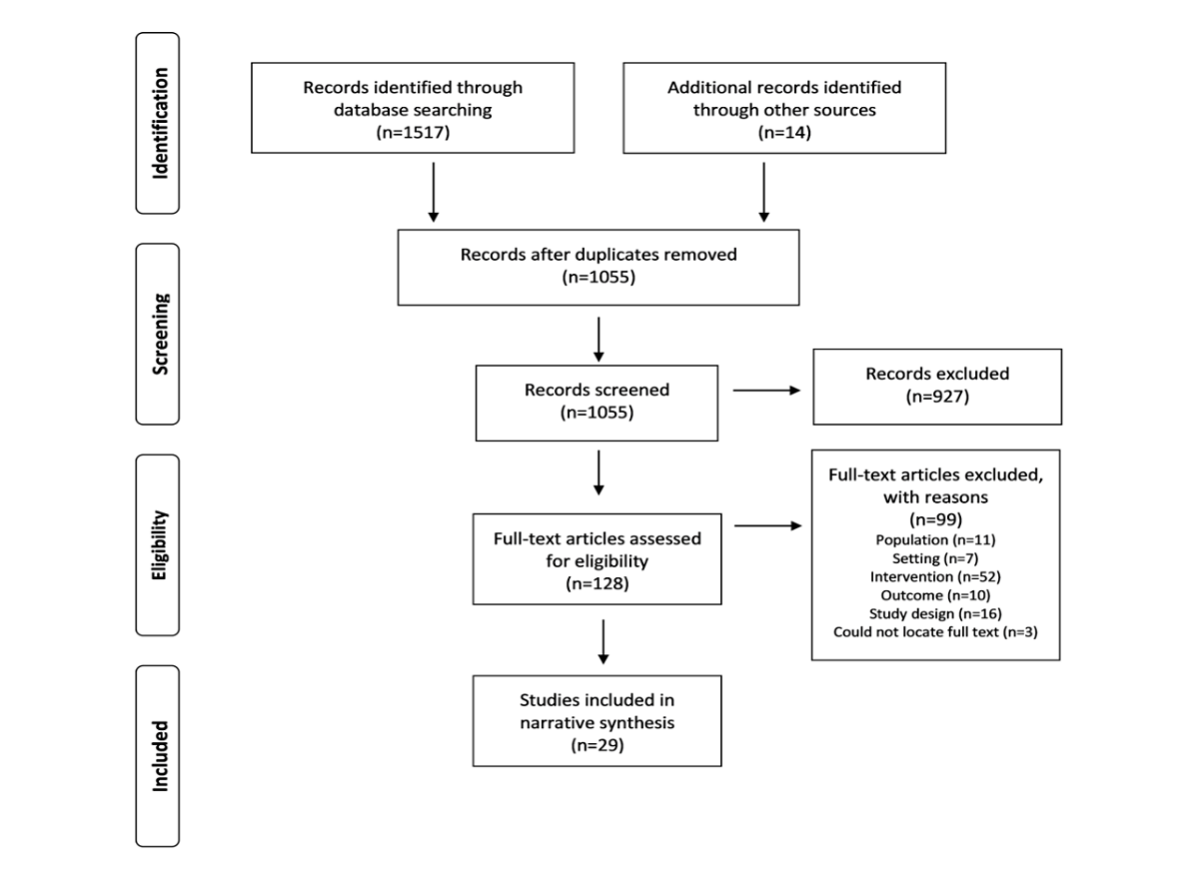
PRISMA (Preferred Reporting Items for Systematic Reviews and Meta-Analyses) flowchart [30].

A total of 1055 papers were screened by title and abstract, of which 128 (12.13%) full-text articles were assessed; of the 128 articles, 29 (22.7%) met the eligibility criteria for the review (Figure 1 [30]). The included studies reported 27 unique interventions that aimed to facilitate comprehensive assessment of care home residents or improve decision-making surrounding care and treatment, published between 2000 and 2020. The sample sizes ranged from 5 to 4171 for residents and 6 to 609 for carers. Approximately 31% (9/29) of studies omitted the number of participants.

Of the 29 articles, the included studies comprised 7 (24%) randomized controlled trials, 7 (24%) quasi-experimental studies [31-37], 6 (21%) qualitative studies [38-43], 3 (10%) descriptive studies [44-46], 2 (7%) mixed methods studies [47,48], 2 (7%) cohort studies [49,50], and 2 (7%) cross-sectional studies [51,52].

All studies included adults with dementia, focusing specifically on requirements for people with dementia (11/29, 38%) [31,32,38-40,43,47,49,50,53,54] or within a mixed population (18/29, 62%) [33-37,41,42,44-46,48,51,52,55-59]. The average percentage of residents with dementia was 52.8% in the mixed population studies that reported the proportion (6/29, 21%) [35,37,44,46,55,56]. Approximately 41% (12/29) of studies did not delineate the proportion of residents with dementia [33,34,36,41,42,45,48,51,52,57-59] (see Table 1 for a summary of study characteristics).

**Table 1:** Summary of characteristics of included studies grouped by population residents.

Characteristics, study, and country of origin		Study design	Population (n)	Age (years), mean	Setting (n)	Type of eHealth intervention
**Population with dementia and specific requirements**						
	Catic et al [49], United States^a^	Cohort	Dementia (46)	82.5	Nursing home (11)	Video consultation
	Gordon et al [50], United States^a^	Matched-cohort study	Dementia (115)	Not specified	Nursing home (11)	Video consultation
	Klein et al [38], Australia	Qualitative	Dementia (5)	59-88	Regional aged care facility (1)	PDA
	Lee et al [53], Korea	Quasi-experimental	Dementia (53)	Not specified	Nursing home (1)	Video consultations and computerized system
	Lyketsos et al [54], United States	Quasi-experimental	Dementia (not specified)	Not specified	Long-term dementia facility (1)	Video consultations
	Mitchell et al [31], United States	Cluster RCT^b^	Dementia (402)	86.7	Nursing home (64)	Video decision support tool
	Qadri et al [47], United States	Mixed methods	Dementia (not specified)	Not specified	Nursing home (3)	PDA
	Moniz-Cook et al [32], United Kingdom	Cluster RCT	Dementia (832)	Not specified	Care home (58)	Computerized decision support tool
	Piau et al [39], France	Qualitative	Dementia (90)	Not specified	Long-term care facility (10)	Video consultations
	Shiells et al [40], Czechia	Qualitative	Dementia (not specified)	Not specified	Nursing home (3)	Electronic patient records
	Keenan et al [43], United Kingdom	Qualitative	Dementia (not specified)	Not specified	Care home (27)	Computerized decision support tool
**Population with mixed requirements (dementia and nondementia)**						
	Salles et al [45], France	Descriptive	Mixed (304); others: wounds and psychiatric	85.6	Nursing home (1)	Video consultations
	Daly et al [33], United States	RCT	Mixed (22)	86	Long-term care facility (1)	Electronic patient records
	De Luca et al [34], Italy	RCT	Mixed (59)	79.1	Nursing home (1)	Video consultations and tele-counseling
	Johnston and Jones [44], United States	Descriptive	Mixed; dementia (52.5%); others: delirium, depression, and dysthymia	79.3	Rural skilled nursing facility (1)	Video consultations
	Krüger et al [55], Norway	Quasi-experimental	Mixed; dementia (76.6%) and stroke (23.4%)	84.4	Nursing home (7)	Electronic patient records with decision support tool
	Mitchell et al [35], United States	Cluster RCT	Mixed; dementia (70%)	Not specified	Nursing home (intervention=119; control=241)	Video decision support tool
	Perri et al [56], Canada	Quasi-experimental	Mixed; dementia (69%); other: cardiovascular, respiratory, frailty, and psychiatric.	87	Long-term care facility (1)	Video consultations
	Alexander [46], United States^c^	Descriptive	Mixed; dementia (20), Alzheimer (13), and other: osteoarthritis, pneumonia, and cerebrovascular accident	Not specified	Nursing home (3)	PDA, electronic patient records, and decision support tool
	Mor et al [37], United States	RCT	Mixed (not specified); dementia (30%); others: cardiopulmonary	Not specified	Nursing home (360)	Video decision support tool
	Vuorinen [41], New Zealand	Qualitative	Mixed (not specified)	≥65 (intervention)	Long-term care facility dementia unit (1)	Web-based assessment tool
	Weiner et al [36], United States	RCT	Mixed (369)	64	Nursing home (1)	Video consultations
	Pillemer et al [57], United States	Quasi-experimental	Mixed (761)	79.4	Nursing home (10)	Electronic patient records and PDAs
	O’Mahony et al [58], United States	Quasi-experimental	Mixed (not specified); dementia, cancer, chronic obstructive pulmonary disease, liver disease, and renal failure	Not specified	Skilled nursing facilities (2)	Video consultations
	Munyisia et al [48], Australia	Mixed methods	Mixed (not specified)	Not specified	Nursing home (1) and specialized home (1)	Electronic patient records
	Alexander et al [42], United States^c^	Qualitative	Mixed (not specified)	Not specified	Nursing home (4)	PDA, electronic patient records, and decision support tool
	Bjarnadottir et al [51], United States	Cross-sectional analysis	Mixed (not specified)	Not specified	Nursing home (927)	Electronic patient records
	Wakefield et al [52], United States	Cross-sectional analysis/longitudinal	Mixed (62); dementia, seizure, Parkinson, and urinary tract infections	72	Long-term care facility (1)	Video consultations
	Fossum et al [59], Sweden	Quasi-experimental	Mixed (491)	84.5	Nursing home (15)	Electronic patient records with decision support tool

^a^Articles from the same study.

^b^RCT: randomized controlled trial.

^c^Articles from the same study.

### Quality Appraisal

The included studies varied in their quality. In general, the CASP criteria for the experimental studies identified consistent reporting of clear and focused aims, with appropriate methodologies to address the research questions. Weaknesses were related to small sample size, evidence of selection and attrition bias, poor description of analysis, and nonblinding. Descriptive studies were generally well-reported (average 86% of MMAT criteria met) and quasi-experimental studies (average 77.7% of JBI criteria met). The weaker study design used mixed methods (average 50% of MMAT criteria met) and cross-sectional studies (average 56.2% of JBI criteria met). Weaknesses pertained to the management of confounding factors and the integration of qualitative and quantitative data. Multimedia Appendix 3 [31-59], details the quality appraisal results for each study using the respective appraisal checklist.

### CFIR Constructs Associated With eHealth Implementation

Table 2 details the respective CFIR constructs and subconstructs that were identified as important determinants for implementation. The number of subconstructs identified per study ranged from 1 to 13, with a median of 6. No major differences in implementation requirements were identified between studies with a specific focus on requirements for people with dementia and studies reporting on dementia within a mixed population (Multimedia Appendix 4). Findings for each CFIR construct and respective subconstructs is presented in turn. Constructs that were identified in ≤2 studies are not presented as a narrative because of insufficient data.

**Table 2:** Factors identified to influence the implementation of eHealth interventions in a care home (N=29).

CFIR^a^ construct and subconstructs				Definition in the context of care homes and integrated care for people with dementia		Total, n (%)
**Intervention characteristics: aspects of eHealth that might affect implementation success**						
	Intervention source		How end users perceive the legitimacy of the eHealth source—whether it has been developed internally as a response to a problem in the care home or externally		0	
	Evidence strength and quality		Stakeholder perception of the strength of the evidence supporting the belief that eHealth will produce the desired outcomes from sources such as published literature		0	
	Relative advantage [36-39,47,48,54-56]		Whether stakeholders perceive eHealth as advantageous over current practice		9 (31)	
	Adaptability [37,40,48,49,52,55,59]		How interoperable eHealth is with current care home information technology systems		7 (24)	
	Trialability		Whether eHealth can be tested initially on a small scale, such as piloted in a small number of care homes		0	
	Complexity [36-38,40,41,44,45,47,48,53,54,56,58]		How simple and user-friendly end users perceive eHealth to be within routine care		13 (45)	
	Design quality and packaging		Stakeholder perception of the physical presentation of the eHealth intervention		0	
	Cost [36,37,47,49-51,53,54]		Cost associated with implementing eHealth		8 (28)	
**Patient needs: the extent to which resident needs are known and prioritized by the care home**						
	Clinical benefit^b^ [34,36,38,39,41,45,47,48,52,55,58]		How clinically beneficial eHealth is perceived to be for the resident		11 (38)	
	Person-Centered care^b^ [40,41,45,52]		Whether eHealth can be tailored to the individual needs of the resident and care home		4 (14)	
	Resident experience^b^ [34,39,40,52,56,57]		The effect that eHealth has on resident needs and satisfaction with care		6 (21)	
**Outer setting: external influences on eHealth implementation**						
	Cosmopolitanism [37,43]		The degree to which the care home is networked with others		2 (7)	
	Peer pressure		Pressure experienced by the care home to implement eHealth		0	
	External policy and incentives [40,49,52-54]		External influences of implementation of eHealth for the care home		5 (17)	
**Inner setting: characteristics of the implementing care home**						
	Structural characteristics [35,37,43,52]		The social architecture, age, maturity, and size of the care home		4 (14)	
	Networks and communications [47]		The nature and quality of social networks and communication within a care home		1 (3)	
	Culture		Norms and values of the care home		0	
	**Implementation climate**		The capacity for change and shared receptivity of individuals to eHealth and the extent to which it will be supported within the care home			
		Tension for change [32]	The extent to which stakeholders perceive current practices as needing change		1 (3)	
		Compatibility [36-38,40,47,51,52,54]	The degree of fit between the care home and eHealth in terms of values and existing workflows		8 (28)	
		Relative priority	Individuals’ shared perception of the importance of implementation within the care home		0	
		Organizational incentives and rewards	Incentives to increase participation with eHealth such as awards and promotions for staff		0	
		Goals and feedback	The degree to which goals of eHealth are acted upon and feedback to staff		0	
		Learning climate [32,33,39,40,43,44,51,52,58,59]	A climate in which staff feel valued in the implementation process and comfortable to participate through encouragement by care home leaders		10 (34)	
	**Readiness for implementation**		Indicators of care home commitment to eHealth implementation			
		Leadership engagement [31,32,37,39,43]	Commitment and involvement of care home managers and leaders in implementation		5 (17)	
		Available resources [32,33,35,36,39,41,42,44,46,48,51-53,59]	The level of care home resources dedicated to eHealth implementation, including money and staff time		14 (48)	
		Access to knowledge and information [31-33,35,37,38,40-44,48,51,52,54,56,59]	Access to sufficient eHealth training for end users		17 (59)	
**Individual characteristics: end-user individual beliefs, knowledge, and attitudes toward eHealth and implementation**						
	Knowledge and beliefs about the intervention [36,38,39,41,43,45-48,51-53,55,56,58,59]		End users’ attitudes toward eHealth and its impact		16 (55)	
	Self-efficacy [33,36,38,41,47,56,59]		End users’ belief in their own abilities to use the eHealth intervention		7 (24)	
	Individual stage of change		The phase an individual is in as they progress toward sustained use of eHealth		0	
	Individual identification with organization		End user’s perception of their relationship with the care home		0	
	Other personal attributes [32,33,40,43,44,58,59]		Individuals’ attributes that affect implementation such as staff willingness, experience, age, or grade		7 (24)	
**Process: stages of the implementation process that can impact its success**						
	Planning [32,38,42-44,46,48,51,54,56-59]		The degree to which tasks required for implementation of eHealth are agreed in advance		13 (45)	
	**Engaging**		Attracting and involving stakeholders in eHealth implementation			
		Champions [32,35,37,43,48,58,59]	Individuals who are dedicated to driving the implementation of eHealth and overcoming resistance in the care home		7 (24)	
		End users^b^ [32,33,37,39,40,42-44,54,59]	Other stakeholders, including end users and staff, within the care home		10 (34)	
		Opinion leaders	Individuals in a care home who have a formal or informal influence on others’ attitudes toward implementation		0	
		Formally appointed internal leaders	Individuals from within the care home who are formally appointed to implement eHealth		0	
		External change agents	Individuals from outside the care home who formally influence implementation of eHealth		0	
	Executing [32,35,37,43,44]		The extent to which eHealth implementation is conducted as planned		5 (17)	
	Reflecting and evaluating [35,37,43,46,51,55,59]		Monitoring of eHealth implementation and feedback about its progress		7 (24)	

^a^CFIR: Consolidated Framework for Implementation Research.

^b^Additional subconstructs identified inductively from the data.

### Intervention Characteristics

This refers to aspects of the eHealth intervention that might affect implementation success in care homes and includes findings relating to intervention complexity, adaptability, and cost.

#### Relative Advantage

eHealth that is perceived as advantageous to an alternative system by improving access to emergency care [39], increasing efficiency [37,38,54,55], and reducing paperwork [47] is more likely to be adopted [48]. Barriers to uptake include a preference for face-to-face consultations [56] and increased time required to organize eHealth consultations [36].

#### Adaptability

eHealth is at an advantage if it aligns with data and technology already in use [37,40,51,52]. Concerns on patient privacy and electronic transfer of confidential information act as *institutional firewalls* [40,49] but can be overcome by encrypting data and assigning residents confidential numbers [49]*.* A way to increase the adaptability of a device is through the provision of customizable tools such as drop-down menus [40]. Incorporating a decision support system in eHealth interventions is advocated to respond to changes in individual residents’ needs by providing alerts and directing staff to appropriate care [40,48,55].

#### Complexity

eHealth is more likely to be implemented if it is straightforward and user-friendly [36-38,40,41,44,45,47,48,53,54,56,58]. Simple devices are regarded as more reliable [36,40,48], and it is recommended that more advanced technology be used only where necessary [36]. Dual systems of paper-based and electronic devices should be avoided to minimize inconsistency [38] and the complexity of data recording [48]. Uptake is also influenced by the ease of access to eHealth [36,37,40,47,54,55], with portable tools improving ease of access [38,48] and thereby saving staff time [37,38,47,55]. However, some staff report that handheld devices are easy to break and misplace [47] and prefer a desktop computer [40]. Technological difficulties, including software and memory issues [47], inability of patients and physicians to see or hear each other [52,56], and difficulty in obtaining technical support despite frequently requiring it [36,56], can impede eHealth implementation [35,36,44,47,53,56,58]. Providing training and specialist technical support for staff are key to overcoming these barriers [40-42,44,46,53,56].

#### Cost

Cost is a major barrier to implementing electronic health records in care homes [52]; for an intervention to be implemented, the benefits must be perceived to outweigh the costs [36,51]. eHealth tools that incur no additional financial cost beyond staff time [37,49] and installation [54] can integrate with preassembled data [37], are cheaper to purchase than full-size computers [47], and are more likely to be adopted. Uptake is optimized by external funding [51,53,54] and intervention outcomes that minimize spending such as reduced resident transition between care settings, for example, unplanned hospitalization [36,49,53], or reduced antipsychotic prescriptions [50].

### Patient Needs

This refers to the extent to which resident needs are known, prioritized, and pursued by a care home. A total of 3 novel subconstructs were identified inductively.

#### Clinical Benefit

An important contributor toward successful implementation is the perceived clinical benefit for residents [34,36,38,39,47,52,55,58]. eHealth interventions are reported to help staff *focus on the resident’s condition* [47], better manage residents’ symptoms and vital signs [34,55,58], improve safety around medication administration [55], enable staff to attend to residents in a timely manner, and identify common behavior patterns [38]. The positive impact of eHealth interventions to minimize burdensome care transitions has also been recognized. Reduced unplanned hospital transitions are suggested to improve residents’ quality of life [45] by preventing disruption in care continuity with attendance to care needs in the care home [38]. Residents appreciate minimal journeys to acute care settings [52] and the benefit of emergency telemedicine sessions to deliver timely skilled health care interventions remotely [39]. Barriers to uptake of eHealth interventions pertained to opinions that the intervention little benefited residents [48] by inadequately monitoring and identifying resident change or deterioration and failing to improve assessment processes [41].

#### Person-Centered Care

Interventions must be tailored to the individual and accommodate changing resident needs if they are to be successfully implemented [40,41,52]. Given the heterogeneous population in care homes and variable incidence of dementia, eHealth interventions will not make a positive difference if they do not have the capacity to assist with dementia-specific needs when required [40,41,45]. Extra consideration must go into ensuring that technology is unobtrusive if used in the presence of this patient group [40,52] who may lack the capacity to understand the change in care. Where residents experience cognitive, visual, or hearing difficulties, eHealth must be tailored, such as through the provision of a larger monitor [52].

#### Resident Experience

A negative resident experience of eHealth obstructs implementation [39,40,57]. Dissatisfaction stems from devices interfering with the time spent with staff [57] and a switch from face-to-face to remote communication [56]. Staff report concerns that using eHealth in the presence of residents may be intrusive and dehumanizing [39,40]. Concerns tend to diminish with continued use of eHealth tools; however, over time, staff acknowledge that eHealth can improve care quality [39,52]. Other residents report no unintentional harm or negative effects on communication [57], with 1 study attributing positive findings to residents, reporting that they felt more *followed and cared for* [34].

### Outer Setting

The outer setting is concerned with external influences on intervention implementation. This was least considered construct across the studies, with the main focus on the ‘External policy and barriers’ subconstruct. External barriers include policies on the medical liability of telemedicine [53], licensing requirements that do not allow physicians to consult across different parts of the country [49], and issues around reimbursement policies [52-54]. External financial support acts as an incentive to circumvent the additional cost barriers to implementation [40,53,54].

### Inner Setting

The most commonly considered construct across the studies was the inner setting, referring to the internal characteristics of a care home. These focused on the 2 main subconstructs of implementation climate (compatibility and learning climate) and readiness for implementation (leadership, available resources, and access to knowledge).

#### Structural Characteristics

The structural characteristics of the care home setting that affect implementation success include the complex patient population, with the heterogeneity of residents’ conditions affecting the compatibility between the intervention and the care home [35,60]. Care home size can also affect the uptake of an eHealth intervention, with larger homes facilitating uptake through more comprehensive provision of information technology services or inhibiting uptake with larger home leaders exhibiting more resistance to adoption and delivering training [43].

#### Implementation Climate

##### Compatibility

Portable eHealth devices that can be used at the point of care [38,40,54] and during nighttime hours [36] prevent disruption to workflow, thereby facilitating uptake [36,37,40,47,51,52,54]. Interventions are at an advantage if their goals are aligned with those of the care home, for example, improving advance care planning [37], and they may face resistance if they do not support existing practice [40]. Providing individually tailored implementation protocols [37] and delivering the intervention when care homes are maximally staffed can ease adoption [43,58].

##### Learning Climate

Creating a climate within the care home that encourages learning and active participation in the intervention is key [32,39,43,44,51,52,59]. Staff members in the frontline must be receptive [43] and participate if they are to influence patient outcomes [33,44] and affect care delivery [32]. Changes to personnel at the site [44] and reluctance from staff [51,52,58,59] undermine a conducive learning climate. Hierarchical staffing can inhibit implementation [32,58] if junior staff feel they are unable to speak up [32] or access the new tools [40,43]. If a learning climate is not fostered, the staff lack practice in delivering an intervention [32] and opportunities to share and embed their learning to change practice [43].

#### Readiness for Implementation

##### Leadership Engagement

Organizational commitment from the top down is crucial [31,32,37,43]. Staff leaders and managers who collaborate with the research team [37] and *lead by example* through attending training and contributing to data collection [43] foster an engaged and cohesive workforce [39]. A lack of enthusiasm from management can lead to delays in implementation and reluctance from other staff [43]. Unwillingness and resistance from general practitioners to change practice also act as a barrier [32,39] and contribute to a fragmented learning climate [43].

##### Available Resources

Providing extra time for implementation planning [33,39,41,46,52] and end-user training [32,33,42,46,52] is required to accommodate a change in practice. A collective staff opinion of insufficient time to adjust to change hinders implementation [32,39,44,48,51,52,59]. Sufficient bandwidth is required to allow eHealth to function properly [35,36,44,53], and insufficient bandwidth can lead to significant *motion artefact* [44] and *jerky motion* images [53]. Additional space may be required for training, either within the home [42] or off site [32]. Equipment needs may include extra computers [42] and software [59], incurring further financial costs [32,51]. Reduced staffing [51], high staff turnover [44,58], and staff absence [43] obstruct the implementation of eHealth interventions. Limited resources can act as a barrier unless benefits can be demonstrated to outweigh costs [43,51]. Managers need to consider the additional resources required to accommodate adoption [43].

##### Access to Knowledge and Information

Providing adequate training to end users is crucial for promoting uptake [31-33,35,37,38,40-44,48,51,52,56,59]. A variety of educational methods are used, including interactive learning strategies [56], lectures, exercises, and group discussions [59]. Training is often provided through a cascaded learning *train-the-trainer* approach [37,43,48]. Preference varies as to receiving training *on the job* [31,40,42] or continuously over a designated period in the months leading up to implementation [35,37,48]. Training tailored to individual needs [38,40,48] can help facilitate uptake, whereas inadequate training can negatively influence perceptions of intervention benefits [32,48] and how staff disseminate knowledge to colleagues [32]. Incorporating a period of joint work and supervision from a qualified external expert can further facilitate the benefits of training [32].

### Individual Characteristics

This pertains to end users’ individual beliefs, knowledge, and attitudes toward eHealth and implementation. The findings focused on 3 main subconstructs.

#### Knowledge and Beliefs About the Intervention

Fostering a positive end-user attitude toward eHealth is key to ensuring willing adoption. eHealth tools that are perceived as high quality [45,48,53] and efficient [38,47,48,55], by cutting back on paperwork [47] and providing instant access to data [48], are more likely to be adopted. Interventions judged to improve staff understanding and knowledge [39,48,52,56,58] and benefit residents [36,39,48,58] are more likely to be used. eHealth devices that improve overall job satisfaction [45,48,55,56] while providing additional benefits, such as reducing staff anxiety [38], and social features that enrich staff lives outside of work [47] encourage use. Conversely, negative attitudes toward eHealth act as barriers to implementation. These include concerns regarding a lack of care improvement [39,41,45,58], a preference to deliver care in person [39,45,52,56], and concerns of inequity in health care provision, resulting in difficulty obtaining family consent [39]. Other barriers include a lack of positive impact on staff workload [36,41,45], concerns over increased time and effort because of implementation [36,39,51,52,58], and uncertainty about the purpose of eHealth and how it works [43,46]. This indicates the need for comprehensive training to highlight the benefits and importance of implementing the intervention [48].

#### Self-efficacy

Self-efficacy refers to an individual’s belief in their own capabilities to implement an intervention. Low self-confidence in ability [47,56,59] and apprehension toward using new technology [38,47] can act as barriers to implementation. These can be overcome by providing sufficient training, which is tailored to individual needs [38,40,48], and on-hand technical support [36,56].

#### Other Personal Attributes

Other issues that affect implementation relate to staff willingness and commitment to ensuring that the intervention is executed as planned [32,33,44,59]. A lack of experience can also act as a barrier if senior members of staff consider more junior members to be less capable of participating [32,40,43,59] because of age, grade, educational background, or experience [32,59]. However, demonstrating that training designed for medical caregivers is effective across disciplines and grades should help dispel these notions [58].

### Implementation Process

This concerned reporting on the stages of the implementation process that affect its success. A novel subconstruct was inductively identified to capture data related to engaging end users and other stakeholders.

#### Planning

Insufficient planning and preparation for change can result in overwhelmed staff perceiving eHealth to be incompatible with their care home [51], technology systems and facilities being inundated [42], and essential care being omitted [46]. Poorly planned study timelines can lead to a low number of participants if enough time is not allocated to recruitment [58] and to implementation impact being undetected if insufficient time is allocated to following up and monitoring outcomes [48,57,59]. Time must also be allocated to prospectively explore different types of eHealth to ensure suitability [54]. Multiple strategies are required to initiate a change in behavior and routine practice [32,38]. Developing contingency plans to support the program [44,54] and establishing a team whose primary goal is to assist implementation can help facilitate uptake [59]. Staff members must be fully informed of the aim of the intervention [43] and have full clarity of their designated role to achieve maximum impact [56].

#### Engaging: Champions

Engaging individuals is key to successful implementation. This is often done through designating *champions*—individuals from within an organization responsible for driving and supporting implementation [32,35,37,43,48,58,59]. To be successful, there must be a sufficient number of assigned champions in each home [32]. They must be committed to the position [43] and not feel undermined by others in leadership roles [32]. Designating champions is often undertaken by the team manager [59] to ensure they are appropriately able to support other staff members [43,48].

#### Engaging: End Users

Engaging champions alone is insufficient; other staff members must be involved for successful implementation [33,37,39,40,42-44,54,59]. For a change in the quality of care to be observed, staff engagement must be sustained [43], with all disciplines, including frontline staff [33,54] and corporate leaders, committed to implementation [37]. Involving end users in the design and development of technology [39,40,59] and considering individual requirements is key if an intervention is to be embedded in a new context.

#### Execution

Low intervention fidelity is a challenge in complex care home settings [35], and residents often do not receive the intervention as planned [32]. Flexible protocols tailored to the care home environment should be developed [37,44]. Barriers to successful execution include lengthy gaps between initial contact with participants and data collection and intervention activity, which can undermine the coherence of the intervention [43].

#### Reflecting and Evaluating

Ongoing evaluation of progress is vital to ensure a new intervention is effectively embedded [37,43,46,51,55,59]. This can be done using routine data collected through the eHealth tool [43], through in-person visits to the home, monthly conference calls, or video status reports [35,37] to determine the effect and use of the intervention [46]. Receiving feedback can also motivate care home participation, with an absence of evaluative feedback leading to a feeling of being *short-changed* [43].

## Discussion

### Principal Findings

**Figure 2:**
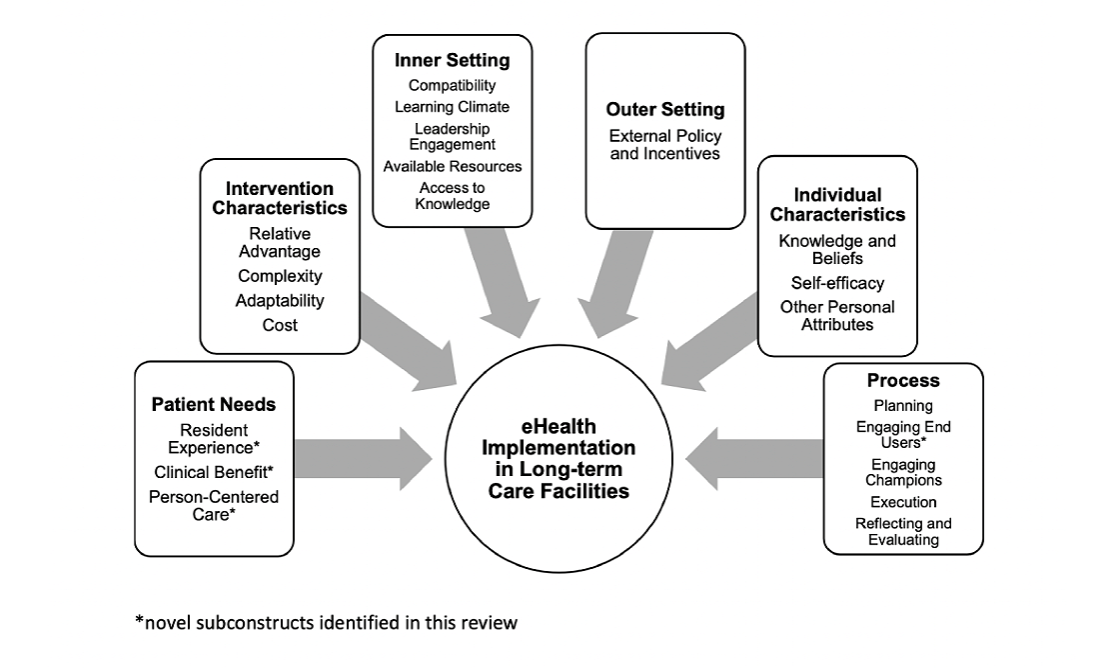
A model of factors that influence the implementation of eHealth in care for people with dementia.

This review investigated the key facilitators and barriers that influence the implementation of eHealth interventions to support comprehensive assessment and decision-making for people with dementia in care homes. Our findings inform a model of eHealth implementation in long-term care for people with dementia (Figure 2). We identified 4 novel subconstructs (denoted in the figure by an asterisk) required to adapt the CFIR to the care home context and use of eHealth to enhance integrated care for people with dementia. This modification enables the framework to accommodate the context-specific findings identified in this review and enhances its use when applied to implement eHealth in the care home context.

The 3 novel subconstructs—*resident experience*, *clinical benefit*, and *person-centered care*—were identified within the *patient needs* construct. No subconstructs have previously been delineated here, and the framework was previously not nuanced enough to capture the data and implementation requirements for this population. *Clinical benefit* was the most commonly identified theme, with the capacity to minimize burdensome transition between care settings as a key facilitator of implementation. This is echoed in the literature that highlights the adverse impact of burdensome care transitions, both on the resident [61] and on costs [62]. Although outcomes were not the focus of this review, eHealth interventions are more likely to be embedded if they can improve the delivery of integrated health care where it is required, enhance integrated care, and improve cost-effectiveness in the National Health Service [63].

Findings around the potential dehumanization of care reiterate a concern raised previously in relation to eHealth implementation [64]. Although the aim of eHealth is to enhance care delivery, entirely omitting in-person contact has clear disadvantages. Striking the right balance between delivering care face to face and virtually is crucial. Future research should focus on delineating the components of health care that must be delivered in person and identifying those that are more amenable to remote delivery to ensure that care quality is not compromised.

The *engaging* subconstruct of the *implementation process* was also modified for the purpose of this review. None of the proposed subcategories of *engaging* (opinion leaders, formally appointed internal implementation leaders, champions, and external change leaders) were suitable for capturing data around engaging end users. The importance of involving end users throughout the implementation process is a key finding and is consistently advocated in the literature to help close the gap between research production and clinical practice [65,66]. Therefore, a novel subcategory titled *end users* was added to the CFIR. Contradictory findings regarding staff and resident preferences on the design and use of eHealth devices are an important indication that there is no *one-size-fits-all* approach to eHealth implementation. This inconsistency in preferences highlights the critical need to involve end users during intervention development to accommodate a range of requirements and enhance the likelihood of sustained implementation. Progressing the CFIR to include a subcategory suitable for data pertaining to all stakeholders’ input increases its relevance and applicability for this context.

The most salient construct identified in this review was *inner setting* of the implementing organization. This is concurrent with the existing research on care homes. A systematic review by Goodman et al [67] identified many of the same factors that influence the readiness of care homes to participate in change. These include ensuring compatibility between the intervention and existing care home routine, providing sufficient training and resources, and engaging care home leaders. Both reviews highlight the significance of the organizational context on implementation success and the need to consider context in the planning stage to inform study design rather than retrospectively. This is contrary to a review of determinants for eHealth implementation with informal caregivers in the community [68], where few factors relating to context were identified as important. This indicates that factors influencing implementation may not be consistent across health care settings and stresses the importance of understanding specific contextual factors and tailoring implementation strategies accordingly.

One of the most prominent determinants of implementation identified in this review was the complexity of the intervention, consistent with previous findings highlighting ease of use as the most important facilitator of eHealth adoption. The nonadoption, abandonment, scale-up, spread, and sustainability framework [69] was developed specifically in response to the finding that eHealth, which is categorized as complex is rarely, if ever, successfully embedded in mainstream care. It aims to help innovators and implementers measure and minimize complexity in eHealth and scale-up and sustain innovation, and therefore, it could helpfully be used in this context.

### Implications for Policy and Clinical Practice

Using the most salient subconstructs of the framework, a conceptual model was developed to highlight the most important factors that influence the implementation of eHealth interventions to enhance integrated care focusing on care processes of comprehensive assessment and decision-making about care and treatment (Figure 2). The findings can be translated into practical recommendations for organizations aiming to embed eHealth within long-term care settings for people with dementia (Textbox 2). The model indicates that for implementation to be successful, eHealth devices must be low cost, simple to use, and tailored to the care home setting and residents. It must be clinically beneficial to the residents, with special consideration of changing, multimorbid dementia-specific needs. Extensive planning and engagement of care home leaders, end users, and champions in the development and implementation process are key to ensuring successful execution. Providing sufficient training and resources to ensure that care home staff feel valued, motivated, and optimistic about a change in practice is crucial to fostering a positive implementation climate.

**Practical recommendation for implementation of eHealth in long-term care for residents with dementia.**

**Consolidated Framework for Implementation Research constructs and practical recommendations for eHealth implementation**

**Patient needs**

- eHealth should be tailored to the individual resident and accommodate changing and complex needs.
- It should be unobtrusive and not replace in-person contact or compromise care quality.
- eHealth must not just streamline workload but clinically benefit the resident and improve outcomes.

**Intervention characteristics**

- eHealth should be user-friendly and accessible to increase sustainability.
- eHealth that is interoperable with current systems is advisable to minimize installation cost and complex new learning for end users.
- Technical support should be easily attainable and readily available.

**Outer setting**

- Policies that endorse eHealth use in care homes ease implementation, for example, rapid policy-driven implementation of remote consultations during the COVID-19 pandemic.

**Inner setting**

- eHealth tools should be tailored to care home settings to fit with existing workflow and care home values.
- Engaging care home leaders in the intervention is key to promoting enthusiasm and a cohesive working environment.
- Staff participation and learning about eHealth should be encouraged.
- Sufficient training should be provided for end users, which should be tailored to individual requirements.
- Additional resources need to be allocated to accommodate a change in practice. These include technological requirements such as bandwidth and equipment and extra staff time.

**Individual characteristics**

- Fostering a positive staff attitude toward eHealth is essential for uptake.
- The tool should benefit staff by easing workload, increasing knowledge, and improving job satisfaction.

**Implementation process**

- Preparation for change and consideration of implementation timelines, strategies, and contingency plans in advance is crucial.
- *Champions* should be designated in each care home to drive and support implementation.
- End users and other stakeholders should be engaged from an early point and consulted in the implementation process to accommodate individual requirements.
- Ongoing evaluation and reflection on uptake and adherence to eHealth should occur to inform any necessary developments and improvements.

### CFIR Framework

The CFIR is a comprehensive determinant framework and is chosen to guide analysis owing to its previous application with eHealth interventions [68,70,71]. Generally, the extracted data were amenable to the CFIR, with no data left uncoded. However, 33% (13/39) of the subconstructs had no associated data (Table 2), concurrent with findings from a previous review of eHealth implementation across health care settings [70]. This consistency either suggests that some subconstructs are not relevant to implementing eHealth or highlights a limitation of the evidence base and lack of existing literature for this setting and population. These areas of uncertainty are important for informing future research, which should focus on identifying barriers to and facilitators of the implementation of these underresearched constructs.

### Strengths and Limitations

A systematic approach to this review allowed for the rigorous and thorough identification of the relevant literature. Evidence synthesis was theory driven and guided by the CFIR, which has been built upon here to increase its relevance in eHealth and long-term care contexts.

Although this review specifies care home residents with dementia as its population of interest, only 38% (11/29) of the included studies had an exclusive dementia population. This was to reflect the real-world heterogeneous populations in care homes and include interventions that were suitable at an organizational level rather than the individual level. Although there was no real difference in factors that influence implementation between dementia-specific and mixed populations, caution must be exercised when extrapolating these findings to homogeneous dementia populations.

Gray literature was not included in this review. This was because, when identified, it provided little data on the factors that influence implementation. This could have excluded relevant data on the eHealth interventions used in care homes. A recent scoping review on telehealth during the COVID-19 pandemic reported a rapid rise in eHealth during the pandemic [72].

### Conclusions

To our knowledge, this is the first review to synthesize evidence on the implementation of eHealth interventions focusing specifically on improving assessment and decision-making in care homes for people with dementia. We adapted the CFIR and progressed its applicability for use in this context. We developed a conceptual model to demonstrate the most important factors to consider when designing and implementing an eHealth intervention and translated it into 18 practical recommendations for implementers, innovators, and organizations to implement eHealth for people with dementia in long-term care. Particular focus should be placed on the individual care home setting and on the consideration of resident and end-user needs when developing an implementation strategy for use in this context.

## Appendix

Multimedia Appendix 1 PRISMA (Preferred Reporting Items for Systematic Reviews and Meta-Analyses) checklist.

Multimedia Appendix 2 Search strategy.

Multimedia Appendix 3 Quality appraisal summary table.

Multimedia Appendix 4 Differences in implementation requirements.

## Abbreviations

CASP: Critical Appraisal Skills Program
CFIR: Consolidated Framework for Implementation Research
ESRC: Economic and Social Research Council
JBI: Joanna Briggs Institute
MMAT: Mixed Methods Appraisal Tool
NIHR: National Institute for Health Research
PRISMA: Preferred Reporting Items for Systematic Reviews and Meta-analyses
PROSPERO: International Prospective Register of Systematic Reviews
