# Supplementary material for: Implementation of eHealth to Support Assessment and Decision-making for Residents With Dementia in Long-term Care: Systematic Review
Source: J Med Internet Res. 2022 Feb 3;24(2):e29837. doi: 10.2196/29837 (PMC8855285; doi:10.2196/29837)
Supplement: Multimedia Appendix 2 [file jmir_v24i2e29837_app2.docx]

**Additional file 2:** Search strategy for CINHAL, MEDLINE, PsycINFO and Embase

| **Database** | **Concept** | **MeSH terms** | **Key words** |
| --- | --- | --- | --- |
| **CINHAL** | Dementia | (MM "Dementia+") OR (MH "Nursing Home Patients") OR (MH "Dementia Patients") | “dementia*” OR “senile dementia” OR “vascular dementia” OR “Alzheimer* or PWD |
|  | Care home | **(**MH "Nursing Homes) OR (MH "Residential Care") | "nursing homes” or “nursing home” or “care home” or “care homes” or “long term care” or “residential care” or “aged care facility" |
|  | eHealth | (MH "Telecommunications+") OR (MH "Telehealth+") OR ("Electronic Health Records") or (MH "Health Information Systems+") | “e#tool” or “e#health” or “digital*” or “health informat*” or “technology*” or “telehealth*” or “telecon*” or “telemed*” or “telediag*” or “telemonitor*” or “telecom*” or “telecare” or “information communication tech*” or “interactive health communication” or “electronic shared record” or “electronic communic*” or "electronic health” or “online*” or “internet” or “video decision support” or “videocon*” or “video-con*” or “mobile* or “m?hteleealth” or “app” or “web*” or “virtual” |
|  | Intervention | (MH "Geriatric Assessment") OR (MH "Clinical Assessment Tools") OR (MH "Decision Making, Clinical") OR (MH "Nursing Process+") OR (MH "Quality of Care Research") | “assessment” or “decision*” or “communicat*” or “intervention” or “delivery of care” or “support” or “advis* or “program” or “educat* or “train*” or “coordinat*” or “integ*” |
| **MEDLINE** | Dementia | exp Dementia/ or Cognitive Dysfunction/ | ("dementia*" or "senile dementia" or "vascular dementia" or "alzheimer*" or "PWD") |
|  | Care home | exp Nursing Homes/ or Homes for the Aged/ or Long-Term Care/ | ("nursing homes" or "nursing home" or "care home" or "care homes" or "long term care" or "residential care" or "aged care facility") |
|  | Telehealth | exp Telemedicine/ or exp Telecommunications/ or Mobile Applications/ or exp Medical Records Systems, Computerized/ | (e?tool or e?health or digital or health informat* or technolog* or telehealth* or telecon* or telemed* or telediag* or telemonitor* or telecom* or telecare or information communication tech* or interactive health communication or electronic shared record or electronic communic* or electronic health or online or internet* or video decision support or Videocon* or video-con* OR mobile* or m?health or app or web* or virtual) |
|  | Intervention | Geriatric assessment/ or Nursing assessment/ or exp Decision Making, Computer-Assisted/ or Internet-Based Intervention/ or "continuity of patient care"/ or Clinical Decision-Making/ | assessment or decision* or communicat* or intervention or care delivery or support or advis* or program or educat* or train* or coordinat* or integ* |
| **PsycINFO** | Dementia | exp dementia/ | dementia* OR senile dementia OR vascular dementia OR Alzheimer* or PWD |
|  | Care home | Long Term Care/ or exp nursing homes/ or residential care institutions/ | nursing homes or nursing home or care home or care homes or long term care or residential care or aged care facility |
|  | eHealth | exp telecommunications media/ or exp telemedicine/ or exp electronic health records/ or exp electronic health services/ or exp videoconferencing/ or exp health information technology/ or exp video based interventions/ | e?tool or e?health or digital or health informat* or technolog* or telehealth* or telecon* or telemed* or telediag* or telemonitor* or telecom* or telecare or information communication tech* or interactive health communication or electronic shared record or electronic communic* or electronic health or online or internet* or video decision support or Videocon* or video-con* OR mobile* or m?health or app or web* or virtual |
|  | Intervention | exp geriatric assessment/ or Neuropsychological Assessment/ or exp "Clinical Judgment (Not Diagnosis)"/ or exp intervention/ or exp "continuum of care"/ | assessment or decision* or communicat* or intervention or care delivery or support or advis* or program or educat* or train* or coordinat* or integ* |
| **Embase** | Dementia | exp dementia/ | dementia* OR senile dementia OR vascular dementia OR Alzheimer* or PWD |
|  | Care home | exp nursing home/ or exp home for the aged/ or exp residential home/ | nursing homes or nursing home or care home or care homes or long term care or residential care or aged care facility |
|  | eHealth | exp telemedicine/ or exp telehealth/ or exp electronic health record/ or web-based intervention/ or exp videoconferencing/ | e?tool or e?health or digital or health informat* or technolog* or telehealth* or telecon* or telemed* or telediag* or telemonitor* or telecom* or telecare or information communication tech* or interactive health communication or electronic shared record or electronic communic* or electronic health or online or internet* or video decision support or Videocon* or video-con* OR mobile* or m?health or app or web* or virtual |
|  | Intervention | Dementia assessment expl/ or exp clinical assessment/ or exp clinical decision making/ or exp intervention study/ | assessment or decision* or communicat* or intervention or care delivery or support or advis* or program or educat* or train* or coordinat* or integ* |
