# Supplementary material for: Implementation of eHealth to Support Assessment and Decision-making for Residents With Dementia in Long-term Care: Systematic Review
Source: J Med Internet Res. 2022 Feb 3;24(2):e29837. doi: 10.2196/29837 (PMC8855285; doi:10.2196/29837)
Supplement: Multimedia Appendix 3 [file jmir_v24i2e29837_app3.docx]

**Additional File 3**: Summary of quality appraisal scores

| **Study design** | **Quality Appraisal Tool** | **Study** | **Score (%)** |
| --- | --- | --- | --- |
| Quasi-experimental | Joanna Briggs Institute (JBI) Checklist for  Quasi-Experimental Studies  (non-randomized experimental studies) | Lee et al, 2000 | 88.8 |
|  |  | Lyketsos et al, 2001 | 22.2 |
|  |  | Kruger et al, 2011 | 77.7 |
|  |  | Perri et al, 2020 | 88.8 |
|  |  | Fossum et al, 2011 | 100 |
|  |  | Pillemer et al, 2012 | 100 |
|  |  | O’Mahoney et al, 2009 | 66.6 |
| Cross- sectional | Joanna Briggs Institute (JBI) Checklist for Analytical  Cross Sectional Studies | Bjarndottir et al, 2017 | 62.5 |
|  |  | Wakefield et al, 2004 | 50 |
| Mixed-methods | Mixed Methods Appraisal Tool (MMAT) | Quadri et al, 2009 | 40 |
|  |  | Munyisa et al, 2011 | 60 |
| Descriptive |  | Johnston et al, 2001 | 60 |
|  |  | Salles et al, 2017 | 100 |
|  |  | Alexander et al, 2008 | 100 |
| Cohort studies | Critical Appraisal Skills Programme (CASP) Cohort Study Checklist | Catic et al, 2014 | n/a |
|  |  | Gordon et al, 2016 | n/a |
| Qualitative | Critical Appraisal Skills Programme (CASP) Qualitative Study Checklist | Klein et al 2018. Australia | n/a |
|  |  | Piau et al, 2020 | n/a |
|  |  | Shiells et al, 2020 | n/a |
|  |  | Vuorinen et al, 2020 | n/a |
|  |  | Alexander et al, 2007 | n/a |
|  |  | Keenan et al, 2020 | n/a |
| Randomised Control Trial (RCT) | Critical Appraisal Skills Programme (CASP) RCT Study Checklist | Mitchell et al, 2018 | n/a |
|  |  | Moniz-Cook et al, 2017 | n/a |
|  |  | Daly et al, 2002 | n/a |
|  |  | De Luca et al, 2015 | n/a |
|  |  | Mitchell et al, 2020 | n/a |
|  |  | Weiner et al, 2003 | n/a |
|  |  | Mor et al, 2017 | n/a |
