# Supplementary material for: Implementation of eHealth to Support Assessment and Decision-making for Residents With Dementia in Long-term Care: Systematic Review
Source: J Med Internet Res. 2022 Feb 3;24(2):e29837. doi: 10.2196/29837 (PMC8855285; doi:10.2196/29837)
Supplement: Multimedia Appendix 4 [file jmir_v24i2e29837_app4.docx]

**Additional File 4:** Differences in implementation requirements between studies focusing specifically on dementia vs. dementia within a mixed population

| **CFIR Construct** | **CFIR Subconstruct** | **% of studies with a specific focus on requirements for people with dementia (n=11)** | **% of studies including dementia within a mixed population (n=18)** |
| --- | --- | --- | --- |
| **Intervention Characteristics** | a) Intervention source | 0% | 0% |
|  | b) Evidence Strength & Quality | 0% | 0% |
|  | c) Relative Advantage | 36% | 28% |
|  | d) Adaptability | 18% | 28% |
|  | e) Trialability | 0% | 0% |
|  | f) Complexity | 45% | 50% |
|  | g) Design Quality & Packaging | 0% | 0% |
|  | h) Cost | 36% | 11% |
| **Patient Needs** | a) Clinical Benefit* | 27% | 39% |
|  | b) Person-centered Care* | 9% | 17% |
|  | c) Resident Experience* | 18% | 22% |
| **Outer Setting** | a) Cosmopolitanism | 9% | 6% |
|  | b) Peer Pressure | 0% | 0% |
|  | c) External Policy & Incentives | 36% | 6% |
| **Inner Setting** | a) Structural Characteristics | 9% | 17% |
|  | b) Networks & Communications | 9% | 0% |
|  | c) Culture | 0% | 0% |
|  | d) Implementation Climate | | |
|  | d1) Tension for Change | 9% | 0% |
|  | d2) Compatibility | 27% | 17% |
|  | d3)Relative Priority | 0% | 0% |
|  | d4) Organizational Incentives & Rewards | 0% | 0% |
|  | d5) Goals and Feedback | 0% | 0% |
|  | d6) Learning Climate | 36% | 28% |
|  | e) Readiness for Implementation | | |
|  | e1) Leadership Engagement | 36% | 6% |
|  | e2) Available Resources | 27% | 61% |
|  | e3) Access to Knowledge & Information | 45% | 61% |
| **Individual Characteristics** | a) Knowledge & Beliefs about the Intervention | 36% | 61% |
|  | b) Self-efficacy | 18% | 22% |
|  | c) Individual Stage of Change | 0% | 0% |
|  | d) Individual Identification with Organization | 0% | 0% |
|  | e) Other Personal Attributes | 9% | 22% |
| **Process** | a) Planning | 36% | 44% |
|  | b) Engaging | | |
|  | b1) Champions | 18% | 22% |
|  | b2) End-users* | 45% | 28% |
|  | b2) Opinion Leaders | 0% | 0% |
|  | b3) Formally Appointed Internal Implementation Leaders | 0% | 0% |
|  | b5) External Change Agents | 9% | 0% |
|  | c) Executing | 18% | 17% |
|  | d) Reflecting & Evaluating | 9% | 33% |

*novel subconstructs identified in this review
